# Bis-Benzylisoquinoline Alkaloids Inhibit Porcine Epidemic Diarrhea Virus In Vitro and In Vivo

**DOI:** 10.3390/v14061231

**Published:** 2022-06-06

**Authors:** Shijuan Dong, Ruisong Yu, Xiaoting Wang, Bingqing Chen, Fusheng Si, Jiaming Zhou, Chunfang Xie, Zhen Li, Daojing Zhang

**Affiliations:** 1 Institute of Animal Husbandry and Veterinary Science, Shanghai Key Laboratory of Agricultural Genetics and Breeding, Shanghai Engineering Research Center of Breeding Pig, Shanghai Academy of Agricultural Sciences (SAAS), Shanghai 201106, China; dsjuan@saas.sh.cn (S.D.); yuruisong@saas.sh.cn (R.Y.); chenbingqing@saas.sh.cn (B.C.); mr.fusheng@saas.sh.cn (F.S.); xiechunfang@saas.sh.cn (C.X.); 2 State Key Laboratory of Bioreactor Engineering, East China University of Science and Technology (ECUST), Shanghai 200237, China; xiaotingwang09@163.com; 3 Shanghai Jinshan District Animal Center of Disease Control, Shanghai 201540, China; 15712642006@163.com

**Keywords:** PEDV, bis-benzylisoquinoline alkaloids, cepharanthine, tetrandrine, fangchinoline, antiviral effect, in vitro, in vivo

## Abstract

Porcine epidemic diarrhea virus (PEDV) belongs to the genus *Alphacoronavirus* of the family *Coronaviridae* that causes severe diarrhea and high mortality in neonatal suckling piglets. Currently, there is no effective medication against this pathogen. Cepharanthine (CEP), tetrandrine (TET), and fangchinoline (FAN) are natural bis-benzylisoquinoline alkaloids with anti-inflammatory, antitumor, and antiviral properties. Here, we first found that CEP, TET, and FAN had anti-PEDV activity with IC_50_ values of 2.53, 3.50, and 6.69 μM, respectively. The compounds could block all the processes of viral cycles, but early application of the compounds before or during virus infection was advantageous over application at a late stage of virus replication. FAN performed inhibitory function more efficiently through interfering with the virus entry and attachment processes or through attenuating the virus directly. CEP had a more notable effect on virus entry. With the highest SI index of 11.8 among the three compounds, CEP was chosen to carry out animal experiments. CEP in a safe dosage of 11.1 mg/kg of body weight could reduce viral load and pathological change of piglet intestinal tracts caused by PEDV field strain challenge, indicating that CEP efficiently inhibited PEDV infection in vivo. All of these results demonstrated that the compounds of bis-benzylisoquinoline alkaloids could inhibit PEDV proliferation efficiently and had the potential of being developed for PED prevention and treatment.

## 1. Introduction

Porcine epidemic diarrhea (PED) is a severe intestinal infectious disease caused by porcine epidemic diarrhea virus (PEDV), leading to acute diarrhea, vomiting, and dehydration in pigs. PEDV belongs to *Alphacoronavirus* in the family of *Coronaviridae* with a single-stranded positive-sense RNA genome encoding four structural proteins (spike, envelope, membrane, and nucleocapsid), two non-structural proteins (pp1a and pp1ab), and an accessory protein (ORF3) [1,2]. The disease can cause 80–100% mortality in newborn piglets [3,4]. Frequent epidemics and endemics of PED have brought heavy economic loss to the world pig industry. Until now, there is no clinically effective medication for the control of this disease.

Natural compounds have been confirmed as being able to curb different kinds of viral pathogens, such as coronavirus, dengue virus, coxsackievirus, and influenza virus [5]. So far, many plant-derived compounds have been screened for antiviral activities against PEDV; for instance, chemical constituents from the leaves of *Sabia limoniacea* were found to have the potential. Among them, Sabphenols A and B showed IC_50_ (half inhibition concentration) values of 7.5 and 8.0 μM, respectively, against PEDV replication [6]. Four oleanane triterpenes (Compounds **6**, **9**, **11**, and **13**) also showed inhibitory effects on PEDV production through downregulating the expression of genes encoding nucleocapsid, spike, and membrane proteins [7]. The polysaccharides isolated from *Ginkgo biloba* exocarp interrupted PEDV attachment and entry to the Vero cells [8]. A recent study on aloe extract has been extended to the stage of animal experiment, in which the extract was shown to protect newborn piglets from lethal challenge [9]. These studies showed the compounds’ promise, but it seems still far for the compounds to be practically used in veterinary clinics. Obviously, more investigations were needed for compound screening and mechanism exploration.

Belonging to the large isoquinoline alkaloid family, bis-benzylisoquinoline alkaloids have various functions of antimicrobials (antiviral [10,11,12], antibacterial [13], antifungal [13]), antiparasitic [14], anti-inflammatory [15,16], and antiallergic [17]. These compounds were found to protect cell lines from the infection of coronaviruses (SARS-CoV, MERS-CoV, and SARS-CoV-2) by blocking virus entry [18]. Among the compounds, tetrandrine (TET), fangchinoline (FAN), and cepharanthine (CEP) exhibited prominent activity against human coronavirus OC43 (HCoV-OC43) [19] and SARS-CoV-2 [20,21,22]. Recently, CEP was identified as the most potent coronavirus inhibitor among the 2406 clinically approved repurposing candidates in a preclinical model [22]. Moreover, studies showed that CEP and FAN inhibited the replication of human immunodeficiency virus type 1 (HIV-1) [11,23] and TET had antiviral effects on dengue [24] and Ebola viruses [12].

Given the reported high activity of bis-benzylisoquinoline alkaloids against viral pathogens, especially coronaviruses, we carried out the present research on CEP, TET, and FAN to explore their anti-PEDV activity. We found high in vitro activity of these compounds against PEDV and proved the antiviral effect of CEP in a PEDV challenge model. The study provided the preliminary data for compounds of bis-benzylisoquinoline alkaloids to be used in PED treatment and anti-PEDV research.

## 2. Materials and Methods

### 2.1. Cells, Virus, and Virus Preparation

Vero CCL-81 cells (African green monkey kidney cells, purchased from ATCC) cells were cultured in Dulbecco’s Modified Eagle Medium (DMEM) (Hyclone, South Logan, UT, USA), supplemented with 10% fetal bovine serum (FBS) (Gibco BRL, Gaithersburg, MD, USA), 100 U/mL of penicillin, and 100 μg/mL of streptomycin, at 37 °C and in a 5% CO_2_–enriched atmosphere. The cell-culture-adapted PEDV DR13^att^ strain (JQ023162; isolated from a commercial vaccine of Green Cross, Yongin, South Korea) and rPEDV-∆ORF3-GFP (recombinant PEDV DR13^att^ whose ORF3 gene is replaced by GFP gene) [25] were propagated in Vero cells with DMEM. Virus titers were determined by using the Reed–Muench method and expressed as tissue culture infective dose 50% (TCID_50_). The PEDV ZJXS11 strain that was isolated from piglet fecal matter in Zhejiang province in 2011 was multiplied in a 1-day-old piglet, whose LD_50_ (the median lethal dose) was determined as 48 copies/mL [26].

### 2.2. Preparation of Compounds

CEP (Energy Chemical, Shanghai, China), TET (J&K, Beijing, China), and FAN (Macklin, Shanghai, China) were dissolved in dimethylsulfoxide (100% DMSO) to make 20 mM stock solutions, stored at −80 °C. The working solution of each compound was diluted to the indicated concentrations with DMEM. Orally administered CEP was suspended with CMC-Na (0.5%).

### 2.3. Compound Cytotoxicity Determination

Determination of compound cytotoxicity was carried out with Vero cells cultured in 96-well plates. The cells were seeded in a volume of 2 × 10^4^ cells/well. When the cells reached 90% confluence in about 24 h, the culture medium was discarded, and the cells were washed three times with PBS. Then the two-fold serial dilutions of the compounds from initial 60 μM were applied to the cells with 6 parallels for each concentration. The cell control and solvent (DMSO) control were set. After interaction for 3 d, the cell viability of each group was detected by using the MTT assay. In brief, 20 μL/well MTT solution (5 mg/mL) was added to the cell sheets and incubated at 37 °C for 4 h. After the incubation, the reaction solution was removed, and 150 μL/well DMSO was added, followed by slow shaking at room temperature for 10 min. The optical density of each well was measured at a wavelength of 490 nm. The relative viability of the cells was calculated based on the following formula: cell survival rate (%) = OD(sample)/OD(control) × 100%, and the half toxic concentration (CC_50_) of the compound was calculated by SPSS 21.0 software(IBM Corporation, Armonk, NY, USA).

### 2.4. Determination of the Half Maximal Inhibitory Concentration (IC_50_) of Compounds

IC_50_ determination of compounds was carried out with Vero cells cultured in 96-well plates. The cells were seeded in a volume of 2 × 10^4^ cells/well. When the cells reached 90% confluence in about 24 h, the cells were infected with PEDV DR13^att^ in multiplicity of infection (MOI) of 0.004 and treated with two-fold serial dilutions of the compounds. The cell control (without virus and compound), virus control (without compound and vehicle), and vehicle (DMSO) control were set. After 3 dpi, the cell viability of each group was determined by using the MTT assay. The relative inhibitory rates of the compound to the virus was calculated based on the following formula: inhibitory rate of the compound to the virus (%) = [OD(sample) − OD(virus control)]/[OD(cell control) − OD(virus control)] × 100%. The IC_50_ of the compound was calculated by using SPSS 21.0 software.

### 2.5. Immunofluorescence Assay (IFA)

For immunofluorescence staining, PEDV-infected cells were washed twice with PBS and fixed with 4% paraformaldehyde for 15 min, followed by membrane permeabilization with 0.1% Triton X-100 in PBS for 15 min at room temperature. After being blocked with 5% bovine serum albumin (BSA), cells were stained with anti-PEDV M polyclonal antibody for 1 h, followed by rinsing three times with PBS and incubation with Alexa Fluor 488-conjugated goat anti-rabbit antibody (Beyotime, Shanghai, China). Nuclei were visualized by using DAPI nuclear. Pictures of immunofluorescent cells were captured using an EVOS fluorescence microscope (M7000, Thermo Fisher Scientific, Waltham, MA, USA) at 100× magnification.

### 2.6. Western Blot Analysis

To detect PEDV N protein expression in PEDV-infected Vero cells, cells were lysed in RIPA lysis buffer with protease inhibitors cocktail (TransGen Biotech, Beijing, China) and subjected to sodium dodecyl sulphate–polyacrylamide gel electrophoresis (SDS–PAGE). SDS–PAGE proteins separated in gel were then transferred to a polyvinylidene fluoride (PVDF) membrane. After protein transfer, the membrane was blocked with 5% non-fat milk in Tris-buffered saline-Tween (TBST) and incubated with anti-PEDV N monoclonal antibody (Shanghai Ango Biotechnology Ltd., Shanghai, China) or anti-GAPDH polyclonal antibody (Sangon Biotech, Shanghai, China) at room temperature for 1 h, and finally incubated with horseradish peroxidase (HRP) conjugated goat anti-rabbit IgG (Sangon Biotech, Shanghai, China). Proteins were detected by using the Amersham ECL Western Blotting Analysis System (GE healthcare, Chicago, IL, USA).

### 2.7. Anti-PEDV IFA and Titration Assays of the Compounds

For analysis of the inhibitory effect of the compounds on PEDV, Vero cells pretreated with the compounds were inoculated with PEDV DR13^att^. The inoculum was kept with cells for 2 h, followed by incubation with fresh maintenance medium containing the compounds. The expression of PEDV M protein in infected cells and the viral titers in supernatant were detected. The exact protocol was as follows.

Vero cells (4 × 10^4^ cells/well) were seeded in 48-well plates. After culture for 24 h, the cells were treated with the compounds at concentrations of 0, 5, 10, and 20 μM for 1 h. Then the cells were inoculated with PEDV DR13^att^. The inoculum was removed after 2 h incubation, and fresh maintenance medium containing 0, 5, 10, and 20 μM compounds was added. Cells were fixed at 24 h post-infection (hpi) and subjected to immune fluorescence staining.

Vero cells (4 × 10^5^ cells/well) were seeded in 6-well plates. After culture for 24 h, the cells were treated with the compounds at concentrations of 0, 5, 10, and 20 μM for 1 h. Then the cells were inoculated with PEDV DR13^att^. The inoculum was removed after 2 h of incubation, and fresh maintenance medium containing 0, 5, 10, and 20 μM compounds was added. Supernatant was collected at 24, 36, and 48 hpi for viral titration (TCID_50_). The cells were collected at 48 hpi and lysed for Western Blot analysis.

### 2.8. To Explore the Time Phase for the Compounds to Inhibit PEDV Replication

Vero cells (2 × 10^4^ cells/well) were seeded in 96-well plates. After culture for 24 h, the cells were treated with the compounds (−1 dpi-tr) at concentrations of 1, 5, 10, and 20 μM. After 24 h, the compounds were removed and washed with PBS three times. The cells were then inoculated with PEDV DR13^att^ for 2 h, followed by 3 d culture. Alternatively, the compounds were applied to the Vero cells at the same time of DR13^att^ inoculation (0 dpi-tr) or 24 h after inoculation (1 dpi-tr) (until the end of test. The GFP fluorescence image was examined at 3 dpi, and the virus inhibition rates of compounds were assessed by using the MTT assay at 4 dpi.

### 2.9. Effects of Compounds on PEDV Administrated in Different Life-Cycle Stage of Virus

Vero cells (4 × 10^5^ cells/well) were seeded in wells of 6-well plates for culture to 90% confluence. The Vero cells were treated with compounds according to the following protocols (For −1 hpi-tr, the cells were pretreated with the compounds CEP, FAN, and TET (20 μM) at 37 °C for 1 h. Then the compound media were removed. The Vero cells were washed twice with PBS and infected with rPEDV-∆ORF3-GFP in an MOI of 0.01. Following a 2 h incubation at 37 °C, the inoculum was removed, and the cells were washed twice with PBS and supplied with fresh DMEM. For 0 hpi-tr, the newly made inoculums from each of the compounds, CEP, FAN, TET (20 μM), and rPEDV-∆ORF3-GFP, were respectively applied to the cells at an MOI of 0.01. Following a 2 h incubation at 37 °C, the inoculums were removed, and the cells were washed twice with PBS for continuous culture in fresh DMEM. For virus-pretr, the above inoculums were preincubated for 1 h at 37 °C before Vero cell inoculation. Following a 2 h incubation at 37 °C with the cells, the inoculum was removed, and the cells were washed twice with PBS for continuous culture in fresh DMEM. For 4 °C Att-tr, Vero cells were inoculated with rPEDV-∆ORF3-GFP at 4 °C for 2 h. Then inoculum was removed, and the cells were washed twice with PBS and incubated with the compounds CEP, FAN, and TET (20 μM), respectively, for 1 h at 37 °C. After the incubation, the compounds were removed, and the cells were washed twice with PBS and supplied with fresh DMEM for continuous culture. For 2 hpi-tr, the protocol was the same as 4 °C Att-tr, except that the inoculation with rPEDV-∆ORF3-GFP was carried out at 37 °C, and the compounds were kept in the culture system to the end of test. Vero cells infected and treated in the above protocols were cultured continuously at 37 °C until 60 hpi. Culture supernatants were collected, and viral titers, as well as mRNA levels of M protein, were detected. Images of cells were captured by using a Zeiss Scope A1 microscope (Zeiss Microsytems, Oberkogen, Germany).

### 2.10. PEDV RNA Extraction and Real-Time RT-PCR

Total RNA was extracted from cells, using the TIANamp virus RNA kit (TIANGEN, Beijing, China) and subjected to reverse transcription with reverse-transcription reagent (Promega, Madison, WI, USA). The standards for SYBR Green real-time RT-PCR were produced by amplification of a 486 bp membrane (M) gene fragment and then cloned into the pJET1.2/blunt vector (Thermo Fisher Scientific, USA) to construct the recombinant plasmids. The primers for standards (sense: 5′-TATTCCCGTTGATGAGGT-3′; antisense: 5′-GCAACCTTATAGCCCTCT-3′) and for qPCR (sense: 5′-TCTTGTGTTGGCACTGTCAC-3′; antisense: 5′-TGCAAGCCATAAGGATGCTG-3′) were synthesized by Sangon Company (Shanghai, China). Real-time RT-PCR was performed on ABI 7500-fast Real-Time PCR systems (ABI, Foster City, CA, USA). Each 20 μL qPCR reaction contained 2 μL reverse-transcription sample, 10 μL TliRNaseH Plus (2×), 0.4 μL forward and reverse primers (10 μM), 0.4 μL ROX Reference Dye II (50×), and 6.8 μL sterile purified water. Amplification conditions were 95 °C for 30 s, followed by 40 cycles of 95 °C for 5 s and 60 °C for 34 s. All samples and standards were carried out in triplicate. The PEDV standards (recombinant plasmid) were diluted serially by tenfold to perform qPCR and establish a standard curve. The concentration of each sample was calculated by blotting Ct values against the standard curve established by serial dilutions of PEDV standards from 10^1^ to 10^8^ copies/μL.

### 2.11. Animal Experiments to Evaluate Anti-PEDV Activity of CEP

Compound CEP was selected for animal experiments to evaluate its anti-PEDV activity. The animal study was approved by the Institutional Animal Care and Use Committee of the Shanghai Academy of Agricultural Sciences (Shanghai, China), and animals were treated in accordance with the regulations and guidelines of this committee.

The toxicity and dosage of CEP were first evaluated in mice. Twenty-five BALB/c mice were purchased from Shanghai SLAC laboratory animal CO., LTD (Shanghai, China), and were randomly divided into five groups (5 mice/group). On day 0, mice in the vehicle group were intragastrically administered with 0.4 mL CMC-Na (0.5%). Mice in groups 1, 2, 3, and 4 were intragastrically administered with 0.4 mL CMC-Na containing CEP at a dose of 100, 500, 1000, and 1500 mg/kg of body weight (bw), respectively. In the acute toxicity study, the death of mice was recorded in 7 days. For the subchronic toxicity assessment, after CEP administration, the body weight of each mouse was measured daily, and all mice were necropsied at 28 days post-inoculation (dpi). At necropsy, blood samples and organ tissue from all mice were collected. The blood samples were examined by blood routine. The pathogenic change of organ tissue was checked, and the viscera index was calculated.

For the antiviral experiment, nine 3-day-old piglets that tested negative for both PEDV RNA and antiserum were divided randomly into 3 groups (3 piglets/group) and were raised in individual rooms. All pigs were artificially fed with milk powder (Beijing Precision Animal Nutrition Research Center, China) during the experiment. After one day of adaptation, the piglets in group 1 (CEP + PEDV) and group 2 (Vehicle + PEDV) were orally challenged with PEDV ZJXS11 (10^2.2^ LD_50_ in 1 mL PBS). Piglets in group 3 (Vehicle) were orally administered the same volume PBS at the same time. When symptoms developed in the challenged animals, group 1 was orally administered CEP compound (11.1 mg/kg in 2 mL CMC-Na, twice a day for three consecutive days). Group 2 and group 3 were orally administered 2 mL CMC-Na twice a day for three consecutive days. Piglets were monitored daily for clinical signs of the disease, rectal temperature, and body weights. Fecal samples were collected at 0, 1, 2, 3, 4, and 5 days post-challenge (dpc), and the virus RNA loads were detected by real-time RT-PCR. After 3 days of continuous administration of CEP, all piglets were sacrificed for tissue collection of duodenum, jejunum and ileum, of which pathological change was also examined by HE staining.

### 2.12. Statistical Analysis

Statistical analyses (one-way ANOVA) were performed by using SPSS software (SPSS 21.0 for windows). Data are expressed as mean values ± standard error (SEM). The *t*-test was employed to determine the statistical significance (*p* < 0.05) of the differences between the means. Data relating to viral RNA copies and virus titer were converted to log10 to maintain a normal distribution.

## 3. Results

### 3.1. The Compounds Diminished Cytopathic Effects of Vero Cells Infected with PEDVs

**Figure 1:**
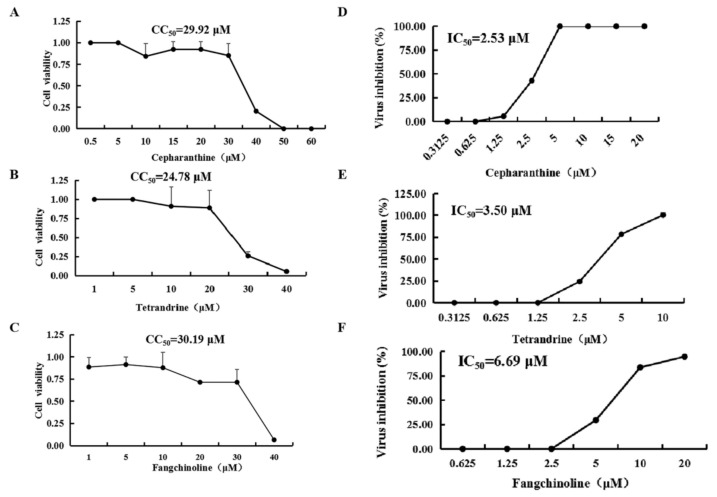
CC_50_ and IC_50_ of bis-benzylisoquinoline alkaloids cepharanthine (CEP), tetrandrine (TET), and fangchinoline (FAN). The confluent monolayer Vero cells were cultured with DMEM medium containing the two-fold serial dilutions of the compounds from initial 60 μM for three days. Cell viability was assessed by using the MTT assay, and the CC_50_ values of the compounds were calculated (**A**–**C**). The confluent monolayer Vero cells were inoculated with PEDV DR13^att^ and treated with two-fold serial dilutions of the compound. After 3 dpi, the cell viability of each group was determined by using the MTT assay, and the IC_50_ values of the compounds were calculated (**D**–**F**). Data are presented as mean ± SEM of three independent experiments.

To measure the cytotoxicity of CEP, TET, and FAN for Vero cells, the CC_50_ values of the compounds were determined by MTT assay. The CC_50_ values of CEP, TET, and FAN were 29.92, 24.79, and 30.19 μM, respectively (Figure 1A–C). For investigating the antiviral effects of CEP, TET, and FAN, the cells were treated with two-fold serial dilutions of the compounds after the cells were infected with PEDV DR13^att^ for 2 h. Three days later, the cytopathic effect (CPE) was obvious in the vehicle-treated Vero cells, but the cells treated with the high-dose CEP, TET, and FAN remained morphologically unchanged. There was variation of CPE with dilutions of the compounds. These results indicated that the three compounds all had potential anti-PEDV activities. The IC_50_ values of CEP, TET, and FAN were 2.53, 3.50, and 6.69 μM, respectively (Figure 1D–F). The selectivity indices (CC_50_/IC_50_) of CEP, TET, and FAN were 11.83, 7.08 and 4.51, respectively, suggesting that CEP had more potential for further in vitro and in vivo tests.

### 3.2. The Compounds Inhibited PEDV Replication in Vero Cells

**Figure 2:**
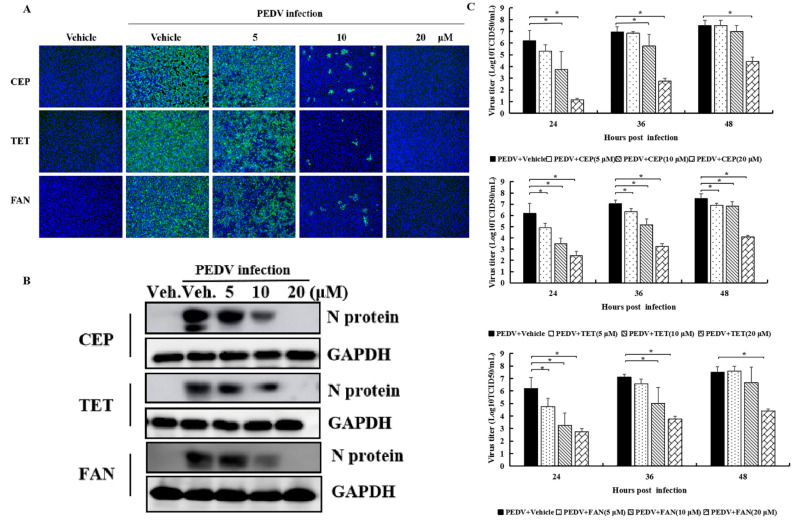
Bis-benzylisoquinoline alkaloids CEP, TET, and FAN inhibit PEDV replication in vitro. Vero cells were treated with 5, 10, and 20 μM of compounds or the solvent (DMSO 0.05%) for 1 h, followed by inoculation with PEDV at an MOI of 0.1. After 2 h, inoculation was removed, and the compounds were applied to the cells again in the same concentrations as above for continuous culture. Vero cells were fixed and subjected to immune fluorescence staining, using anti-PEDV M polyclonal antibody at 24 h post-inoculation (**A**). The cell lysates were prepared to examine PEDV N protein expression with Western Blot (**B**). Vero cells were treated as described above, and supernatant was collected at 24, 36, and 48 hpi for viral titration (TCID_50_) (**C**). (Vehicle: 0.05% dimethyl sulfoxide (DMSO) as solvent for the compounds). Data are presented as mean ± SEM of three independent experiments; * *p* < 0.05.

To further investigate the antiviral effects of the compounds in PEDV-infected cells, IFA of M protein and Western Blot analysis on N protein of PEDV were carried out. The compounds were kept in a culture system after virus inoculation. As shown in Figure 2A, with increasing concentrations of CEP, TET, and FAN, the M protein fluorescence gradually reduced in the cells, indicating a dose-dependent replication inhibition of CEP, TET, and FAN on PEDV. As the concentration of compounds reached 20 μM, no fluorescence image was observed in infected cells, indicating a possible complete inhibition of PEDV. To further prove the inhibitory effect of the compounds on PEDV, N protein expression was analyzed with Western Blot with similarly treated Vero cells. Consistently with the M protein, the expression of N protein proportionally decreased in infected cells as the concentration of compounds increased (Figure 2B). As the concentration of compounds reached 20 μM, no N protein expression was detected in infected cells. The titration results were similar. As shown in Figure 2C, the three compounds with a concentration of 20 μM could significantly inhibit replication of PEDV at different time points compared to the control. In brief, CEP, TET, and FAN could inhibit PEDV replication in vitro.

### 3.3. Compounds Produced Different Inhibitory Effect to PEDV at Different Administration Time

**Figure 3:**
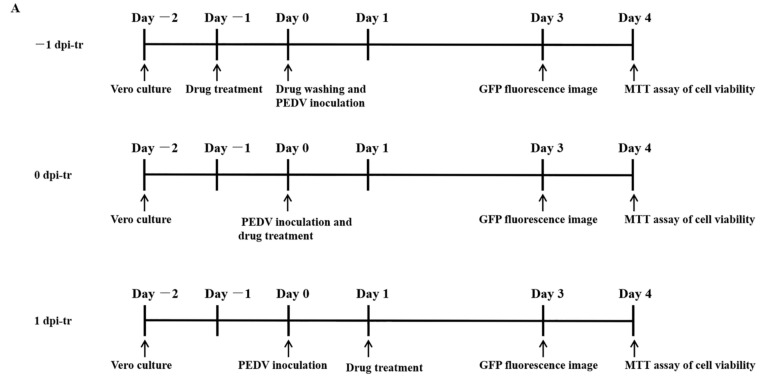

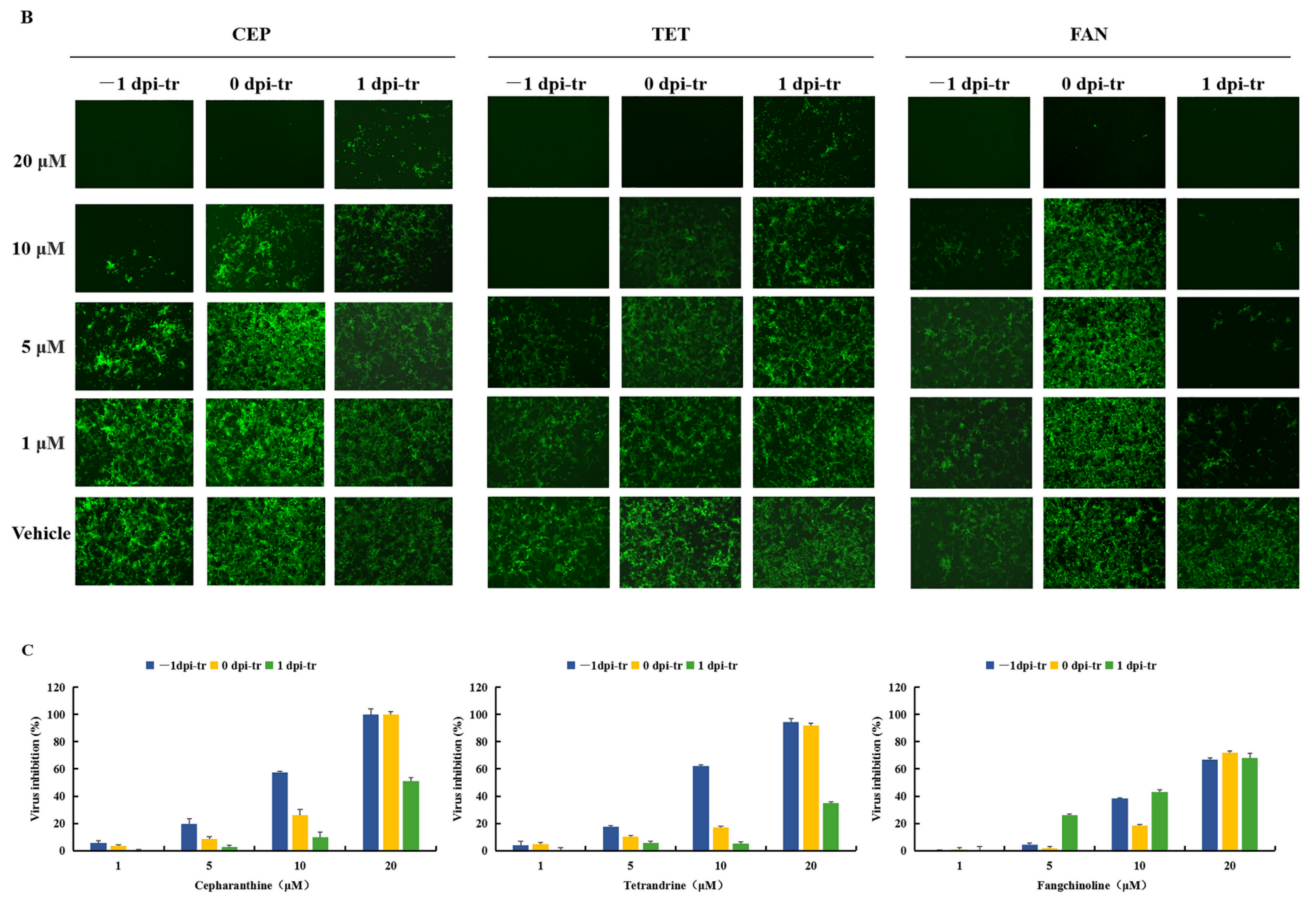
Dose- and time-dependent antiviral activities of CEP, TET, and FAN. Vero cells were inoculated with rPEDV-∆ORF3-GFP, with the addition of TET, FAN, and CEP, at the indicated concentrations or with addition of the vehicle (0.05% dimethyl sulfoxide (DMSO)). The compounds were added at 24 h before, during, or 24 h after PEDV inoculation of Vero cells. (**A**) Time schedules of compound treatments and assay operations. (**B**) GFP expression images of rPEDV-∆ORF3-GFP with different treatments indicated. (**C**) Inhibition rates of virus replication after different treatments indicated. Data are presented as mean ± SEM of three independent experiments.

To know the proper time phase for the compounds to inhibit virus replication, they were applied to the cells at different time points of the PEDV infection. The first was pretreatment, in which Vero cells were treated with different concentrations of the compounds for 24 h before infection with PEDV (−1 dpi). Titration was measured at 3 dpi. The second was the co-treatment, in which compounds were applied to the Vero cells during virus inoculation (0 dpi). The last was post-treatment, in which the compounds were applied 24 h after virus inoculation (1 dpi) (Figure 3A). As shown in Figure 3B, a higher concentration of compounds produced more efficient inhibition to the virus replication. For CEP and TET, pretreatment and co-treatment generated more efficient inhibition on virus replication than post-treatment (Figure 3C). However, for FAN, the virus inhibition rates of three treatments were similar in high concentration (20 μM) (Figure 3C). In lower concentrations of FAN, the post-treatment and pretreatment were also similar, but they were more efficient than the co-treatment. Among the three compounds, the virus inhibition rates of FAN were generally lower than those of CEP and TET.

### 3.4. The Compounds Inhibited Pedv Replication More Efficiently during Early Stages of the Virus’s Life Cycle

**Figure 4:**
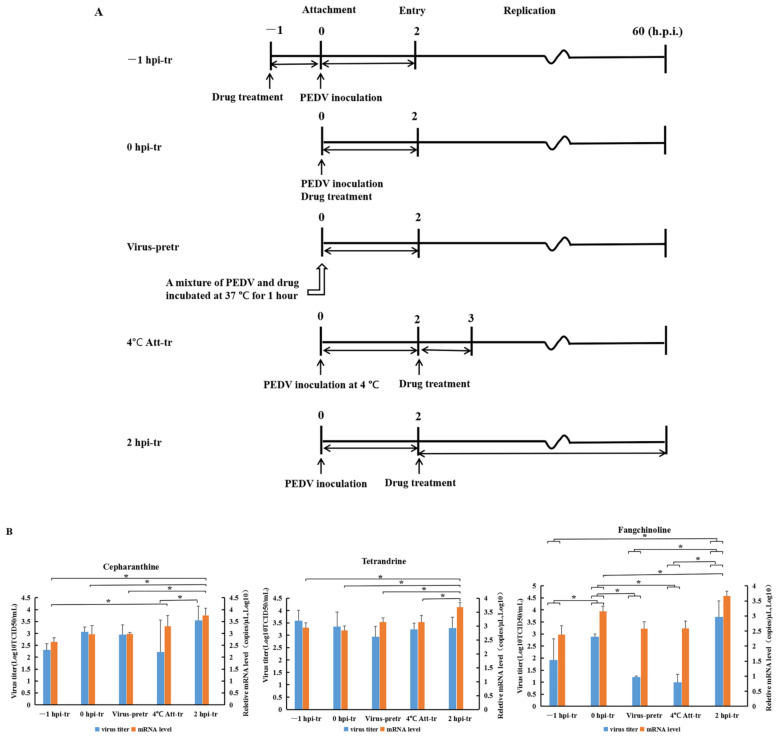
Titers and M protein mRNA level of rPEDV-∆ORF3-GFP after compound treatments at different stages of virus life cycle. (**A**) Time schedules of compound treatments and assay operations. Only attachment, entry, and replication stages of viral life cycle were indicated in the figure for easy illustration. In the treatment of −1 hpi-tr, cells were treated with the compounds 1 h before virus inoculation. In the 0 hpi-tr, compounds were applied to cells at the same time of virus inoculation. In virus-pretr, the viruses and compounds were incubated for 1 h at 37 °C first, and then the mixture was added to cells for inoculation. In 4 °C Att-tr, the viruses were inoculated to cells at 4 °C and kept in this condition for 2 h; then the inoculation was discarded, and the culture temperature was raised to 37 °C for drug treatments and further culture. In 2 hpi-tr, the compounds were applied after 2 h of virus inoculation. (**B**) The 60 hpi titrations and mRNA expression of M protein of rPEDV-∆ORF3-GFP with different treatment protocols. Data are presented as mean ± SEM of three independent experiments; * *p* < 0.05.

As shown in Figure 4A, experiments were carried out to know at which stage of the virus life cycle the compounds perform their function most efficiently. The protocols of the compounds’ administration and the viral-cycle stages possibly blocked were as follows: for −1 hpi-tr, attachment; for 0 hpi-tr, attachment and entry; for virus-pretr, interaction of compounds and virus; for 4 °C Att-tr, entry; and for 2 hpi-tr, virus replication. The administration of compounds (20 μM) at any stage could inhibit the virus, as reflected by the significantly lower mRNA level of M protein and viral titers comparing the vehicle treatment sample (*p* < 0.05) (Supplementary Figure S1). From Figure 4B, it could be found that PEDV M protein expression was higher in 2 hpi–treatment cells than most of the other treatments, and this indicated that later treatment with any of the compounds was not as efficient as early treatments for curbing the expression of viral structural protein. The results also showed that treatment with CEP and FAN after inoculation of viruses at 4 °C could inhibit the PEDV strain most efficiently. For FAN, virus titers of −1 hpi-tr and virus-pretr groups were significantly lower than 0 hpi-tr and 2 hpi-tr groups (*p* < 0.05), indicating that these two kinds of treatments also produced considerable inhibition on virus replication. There was no big difference of viral titers with the different TET treatments (*p* ≥ 0.05). From the above results, it could be concluded that administration of compounds at an early stage of viral cycle could generate more efficient results.

### 3.5. CEP Protects Piglets against PEDV Infection In Vivo

Due to the highest SI index of CEP among the compounds, CEP was chosen for the animal experiment. Before the piglet experiment, the mice were used for a toxicity evaluation. The mice were orally administered CEP at dosages of 0, 100, 500, 1000, and 1500 mg/kg of body weight. The survival rate, body weight, blood physiological index, and viscera index of the mice in all dosage groups were evaluated. The related data are presented in Supplementary Figure S2 and Table S1. The results showed that the oral administration of 100 mg/kg CEP in mice was safe. The safe dosage of mice was converted to the data for piglets by using the equivalent dosage-conversion table [27,28], obtaining the safe dosage of 11.1 mg/kg for newborn piglets.

**Figure 5:**
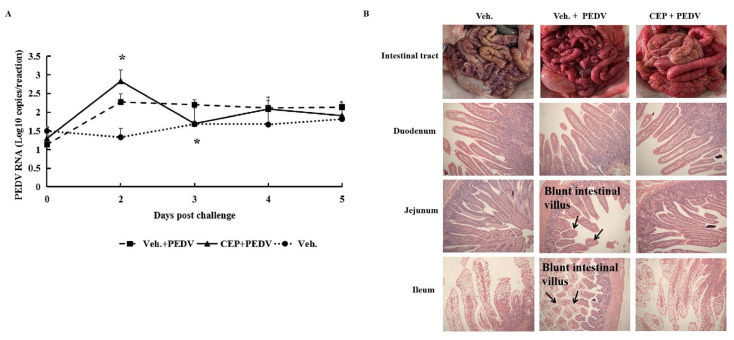
Viral genome RNA loads and intestine pathological change of piglets challenged with wild-type PEDV and treated with CEP. (**A**) The fecal RNA loads of challenged PEDV in the groups of piglets. Data are presented as mean ± SEM; * *p* < 0.05. (**B**) Macroscopic pictures of intestine and HE-stained duodenum, jejunum, and ileum tissue section of piglets from different groups at the 3rd day of CEP administration. Blunt intestinal villus is indicated by arrows. Piglets in vehicle group were not challenged and were administrated with vehicle (vehicle: CMC-Na (0.5%)) as normal control.

The challenge with the virulent PEDV led to mild diarrhea or semi-watery diarrhea in the piglets of group one and group two at 41 hpc. There were no PED symptoms with the piglets in the vehicle group. Following the onset of symptoms, the piglets in group one were orally administrated CEP at a dosage of 11.1 mg/kg twice a day for 3 consecutive days. Animals in group two were given the vehicle, CMC-Na. One day after administration (3 dpc), the viral load of the treatment group was significantly lower than that of the untreated group, demonstrating an inhibitory effect of CEP on PEDV replication in vivo (Figure 5A). All piglets in the three groups were euthanized on the 3rd day of compound administration for tissue lesion check. No lesions of the intestinal tract were observed in any of the groups. However, microscopic pathology section showed mild-to-moderate villous shortening, blunting, and abruption on the jejunum and ileum tissues of the piglets challenged but receiving no CEP treatment (Figure 5B), while the jejunum remained normal in the normal control and CEP-treatment groups. These results confirmed that CEP could protect piglets against PEDV infection in vivo.

## 4. Discussion

CEP, TET, and FAN belong to bis-benzylisoquinoline alkaloids, which possessed not only anticancer and anti-inflammatory activities but also antiviral activities [29]. For instance, CEP, TET, and FAN produced IC_50_ value of 0.33, 1.01, and 0.83 μM on HCoV-OC43 [19]. In our study, we first found that CEP, TET, and FAN imposed a strong inhibitory effect on PEDV in vitro, with IC_50_ values of 2.53, 3.50, and 6.69 μM, respectively, reconfirming their inhibitory effect on coronaviruses. Among them, CEP exhibited the highest SIs (selective indices) of 11.83. We also found that 10~20 μM CEP, TET, and FAN had their proportionally inhibitory effect on PEDV, and 20 μM compounds could almost totally inhibit the PEDV strain in vitro.

In order to investigate the function mechanism of the compounds, we carried out the in vitro application study on different times of virus infection. A previous study on these compounds showed that MRC-5 cell survival rate after HCoV-OC43 infection was higher by pre- and co-treatment of the compounds than post-treatment [19]. In our present study, CEP and TET also had good function through both pretreatment and co-treatment. For FAN, however, virus inhibition rates were more or less similar for three treatments in high concentration, but the inhibition rates were generally lower than CEP and TET. The result suggested a possible better function of CEP or TET on PEDV and earlier viral cycle administration for more efficient function.

To further investigate at which stage of the viral replication cycle the compounds perform their function, experiments were carried out in the present study as to the key time points or elements of the viral cycle. The tests with CEP and FAN showed the inoculation of the virus at 4 °C for 2 h, followed by compound treatment, could inhibit the PEDV strain most efficiently (Figure 4B). The result indicated that the two compounds might perform a more efficient function at the virus entry stage. A recent study reported similar function of CEP on SARS-CoV-2, whose entry of host cells was blocked by the compound [21,30]. CEP was also found to have a potent inhibitory activity against pangolin coronavirus GXp2V, a SARS-CoV-2-related coronavirus, at both the entry and post-entry stages [20]. There was also a report that TET had anti-SARS-CoV-2 activity through inhibiting TPC2 (two-pore cation channels), which is involved in the viral entry process through endosome [31], but we did not observe the same effect of TET on PEDV in the present study. Maybe the difference in the result aroused from the different testing system and the viruses. It is obvious that more studies are needed to confirm this mechanism.

Besides interfering virus entry, the compounds may also attenuate the virus and interfere with other processes of the viral cycle, as all the treatments decreased the mRNA level of M protein and viral titers (Supplementary Figure S1). No doubt, the process that draws attention next is the attachment. For FAN, both the mRNA level and viral titers in the −1 hpi-tr group were significantly lower than the 0 hpi-tr and 2 hpi-tr groups (*p* < 0.05) (Figure 4B). The −1 hpi-tr group was designed to check whether the pretreatment of the cells with the compounds might interfere with the virus attachment process. Thus, logically, the results indicated that FAN might interfere with virus combining to cell receptors. In the 0 hpi-tr group, the compounds would combine or react with the viruses and cells at the same time, thereby interfering with the interaction of the S protein and receptors. Although greater influence was found with this treatment than the 2 hpi- tr from the mRNA level of M protein, the greater influence was not observed from virus titer level. In the virus-pretr group, the compound might change the biochemical nature of S protein or other virus structural proteins, the result of which was a kind of attenuation for the viruses. It is reported that the CEP molecule could bind to SARS-CoV-2 spike protein and interfere with the spike engagement to its receptor [30]. Another research study reported that polysaccharide from *Ginkgo biloba* exocarp can totally inactivate PEDV when treated at 37 °C for 1 h with a concentration of 10 μg/mL [8]. For FAN, both the mRNA level and viral titers in the virus-pretr group were significantly lower than the 0 hpi-tr and 2 hpi-tr groups (*p* < 0.05) (Figure 4B), indicating that the pretreatment might attenuate the viruses.

Although late-stage administration (2 hpi-tr) of the compounds in the research was not the most efficient way to curb virus infection and replication, it is a promising way, considering that the time of its administration is behind virus infection. Compounds may penetrate into cells in this circumstance and work on biological processes involving virus replication. Recently there was a report that aloe extract exerted its inhibition on PEDV at the late stage of the viral life cycle [9]. CEP was also shown suppressing herpes simplex virus type 1 replication through the downregulation of the PI3K/Akt and p38 MAPK signaling pathways [32]. No doubt, the later administrated compound could also block entry and attachment of viruses when the viruses underwent second or more rounds of replications.

As is well-known, the function of medicine in vitro is not equal to the function in vivo. With a high SI index of 11.83, CEP was chosen in the study to explore the anti-PEDV activity in piglets. The results of treatment experiment with the challenged piglets showed that the viral load of CEP-treatment animals was significantly lower than that of the control and the intestinal villi of treatment animals were more intact. The results further proved the PEDV inhibitory function of CEP in vivo, which demonstrated the possibility of application of bis-benzylisoquinoline alkaloids for PEDV treatment in veterinary clinic.

## 5. Conclusions

This study investigated the anti-PEDV effects of the bis-benzylisoquinoline alkaloids CEP, TET, and FAN and determined their working efficacy (IC_50_) with values of 2.53, 3.50, and 6.69 μM, respectively. The compounds could block all the processes of viral cycles, but early application of the compounds before or during virus infection was advantageous over application at a late stage of virus replication. FAN performed the function more efficiently through interfering with the entry, attachment processes, or through attenuation of the virus directly. CEP presented higher inhibition efficiency on virus entry than on other processes. In vivo anti-PEDV activity of CEP was also confirmed with the piglet model. These findings provided preliminary data for the prevention and curing research on PEDV with plant-derived compounds.

## Data Availability

Not applicable.

## Supplementary Materials

The following supporting information can be downloaded at https://www.mdpi.com/article/10.3390/v14061231/s1. Figure S1: Titers and M protein mRNA level of rPEDV-∆ORF3-GFP after compound or vehicle treatments at different stages of virus life cycle. Figure S2: Toxicity determination of CEP in mice. Table S1: Organ indexes of mice administrated different concentrations of CEP.
