# Supplementary material for: Bis-Benzylisoquinoline Alkaloids Inhibit Porcine Epidemic Diarrhea Virus In Vitro and In Vivo
Source: Viruses. 2022 Jun 6;14(6):1231. doi: 10.3390/v14061231 (PMC9228057; doi:10.3390/v14061231)
Supplement: Supplementary file 1 [file viruses-14-01231-s001.zip › supplementary materials.pdf]

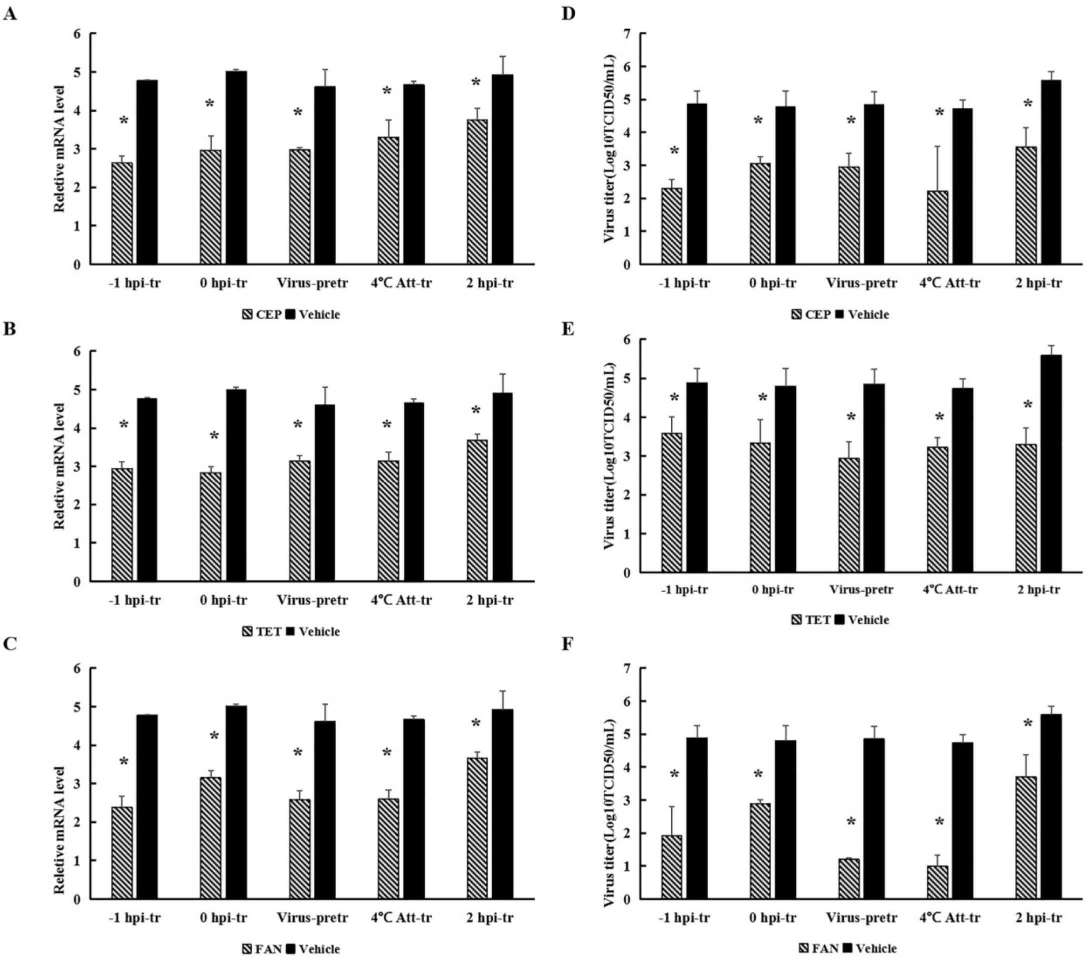


**Figure S1**. Titers and M protein mRNA level of rPEDV-ΔORF3-GFP after compound or vehicle treatments at different stages of virus life cycle. In -1 hpi-tr, cells were treated with the compounds or vehicle 1h before virus inoculation. In the 0hpi-tr, compounds or vehicle were applied to cells at the same time of virus inoculation. In virus-pretr, the viruses and compounds or vehicle were incubated for 1h at 37℃ first, then the mixture was added to cells for inoculation. In 4℃ Att-tr, the viruses were inoculated to cells at 4℃ temperature and kept at this condition for 2h, then the inoculation was discarded and culture temperature raised to 37℃ for drug or vehicle treatments and further culture. In 2hpi-tr, the compounds or vehicle were applied after 2h of virus inoculation. (A-C) M protein mRNA level of rPEDV-ΔORF3-GFP at 60h.p.i with treatments of CEP, TET, FAN or vehicle. (D-F) Titers of rPEDV-ΔORF3-GFP at 60h.p.i with treatments of CEP, TET, FAN or vehicle. Vehicle: 0.1% dimethyl sulfoxide (DMSO). Data are presented as mean±SEM of three independent experiments. * $p < 0.05$.

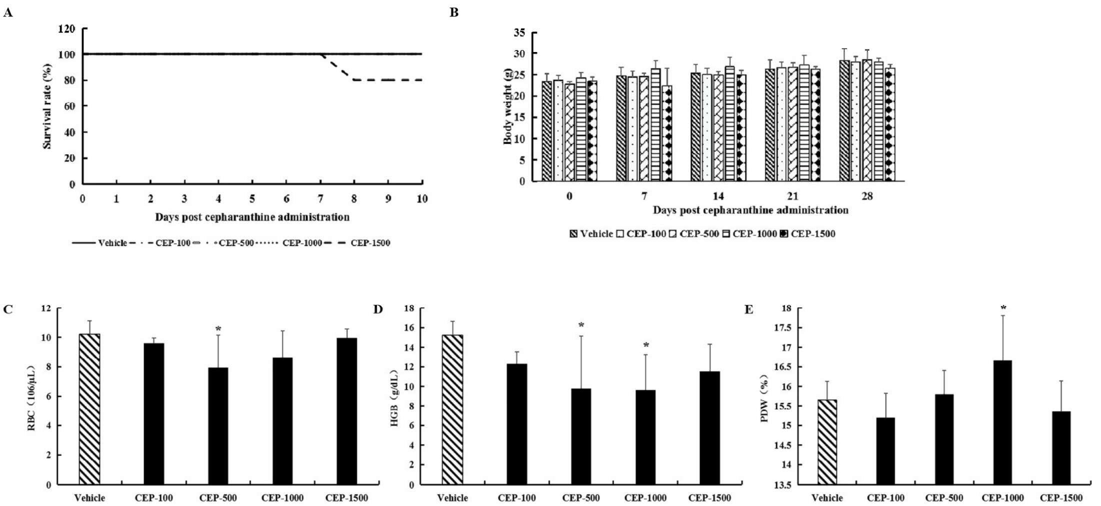


**Figure S2**. Toxicity determination of CEP in mice. Mice were orally administered 0.4ml CMC-Na (0.5%, Mock) or CEP in a single dose of 100(CEP-100), 500(CEP-500), 1000(CEP-1000) and 1500 mg/kg of body weight (CEP-1500) and survival curves (A) and growth of body weight (B) of the mice were recorded at different time after drug or vehicle administration. Mice were sacrificed at 28d of drug administration and red blood cell counts (RBC) (C), Hemoglobin (HGB) (D) and platelet distribution width (PDW) (E) were detected. Data are presented as mean±SEM. * $p < 0.05$.

**Table S1.** Organ indexes of mice administrated different concentrations of CEP.

| Group | | Spleen index（%） | Lung index（%） | Kidney index（%） | Liver index（%） |
|---|---|---|---|---|---|
| Vehicle | | 0.44±0.09 | 0.70±0.15 | 2.14±0.29 | 6.00±0.58 |
| Cepharanthine | 100 mg/Kg | 0.43±0.09 | 0.64±0.09 | 1.93±0.18 | 5.63±0.38 |
| | 500 mg/Kg | 0.40±0.07 | 0.59±0.02* | 2.07±0.20 | 6.26±0.28 |
| | 00 mg/Kg | 0.40±0.08 | 0.60±0.03 | 1.86±0.11* | 5.65±0.35 |
| | 1500 mg/Kg | 0.41±0.06 | 0.66±0.09 | 2.03±0.09 | 5.59±0.26 |

Mice in vehicle group were each administrated 0.4ml CMC-Na(0.5%).
