# Supplementary material for: Bis-Benzylisoquinoline Alkaloids Inhibit Porcine Epidemic Diarrhea Virus In Vitro and In Vivo
Source: Viruses. 2022 Jun 6;14(6):1231. doi: 10.3390/v14061231 (PMC9228057; doi:10.3390/v14061231)
Supplement: Supplementary file 1 [file viruses-14-01231-s001.zip › Table S1.pdf]

**Table S1.** Organ indexes of mice administrated different concentrations of CEP.

| Group | | Spleen index（%） | Lung index（%） | Kidney index（%） | Liver index（%） |
|---|---|---|---|---|---|
| Vehicle | | 0.44±0.09 | 0.70±0.15 | 2.14±0.29 | 6.00±0.58 |
| Cepharanthine | 100 mg/Kg | 0.43±0.09 | 0.64±0.09 | 1.93±0.18 | 5.63±0.38 |
| | 500 mg/Kg | 0.40±0.07 | 0.59±0.02* | 2.07±0.20 | 6.26±0.28 |
| | 00 mg/Kg | 0.40±0.08 | 0.60±0.03 | 1.86±0.11* | 5.65±0.35 |
| | 1500 mg/Kg | 0.41±0.06 | 0.66±0.09 | 2.03±0.09 | 5.59±0.26 |

Mice in Vehicle group were each administrated 0.4ml CMC-Na(0.5%).
