# Supplementary figures and images for: Bis-Benzylisoquinoline Alkaloids Inhibit Porcine Epidemic Diarrhea Virus In Vitro and In Vivo
Source: Viruses. 2022 Jun 6;14(6):1231. doi: 10.3390/v14061231 (PMC9228057; doi:10.3390/v14061231)

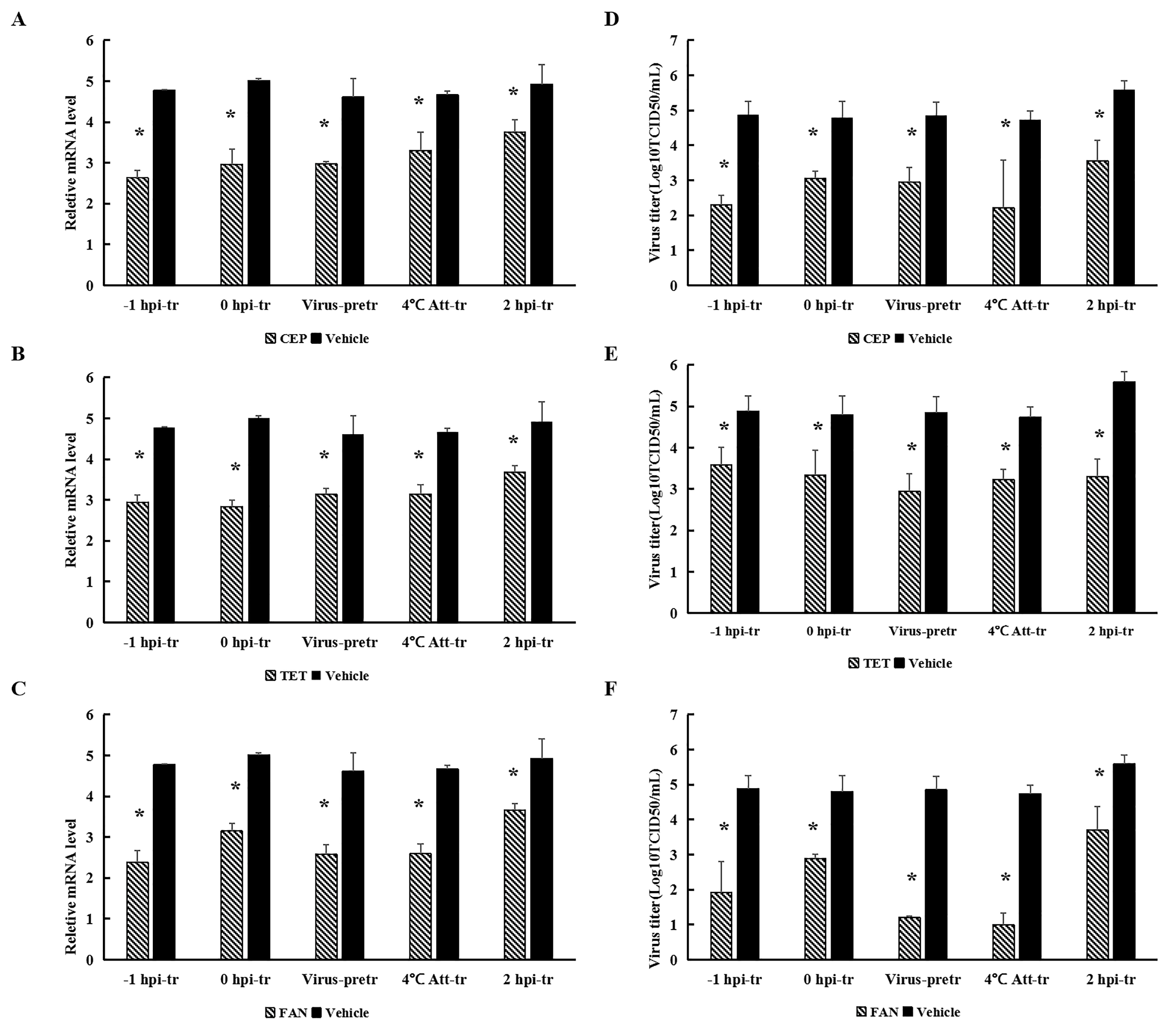

Supplement: Supplementary file 1 [file viruses-14-01231-s001.zip › Figure S1.png]

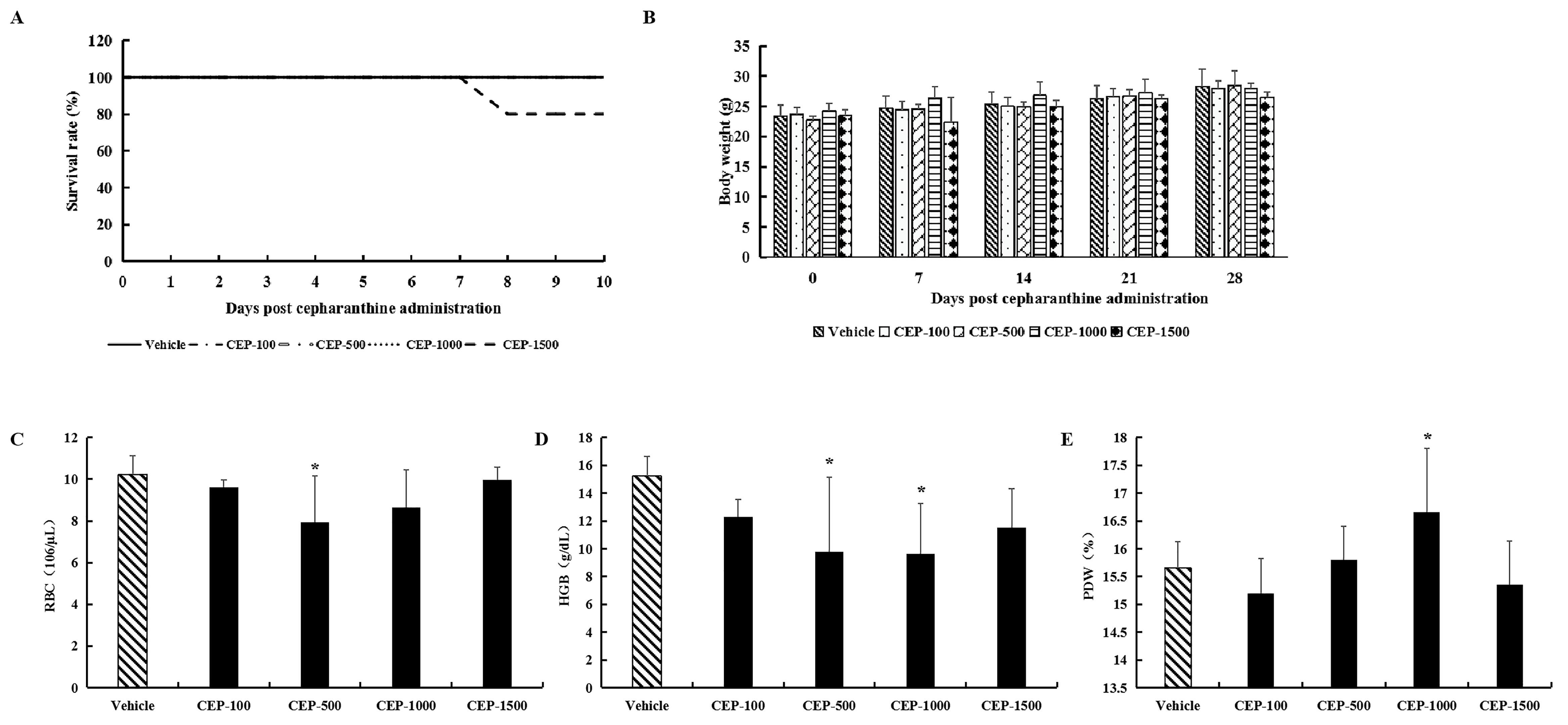

Supplement: Supplementary file 1 [file viruses-14-01231-s001.zip › Figure S2.png]
